# Can Virtual Reality Help Improve Motor and Cognitive Function in Active Aging in Older Adults? A Scoping Review

**DOI:** 10.3390/healthcare12030356

**Published:** 2024-01-30

**Authors:** Víctor Ortiz-Mallasén, Eloy Claramonte-Gual, Víctor Manuel González-Chordá, Irene Llagostera-Reverter, María Jesús Valero-Chillerón, Águeda Cervera-Gasch

**Affiliations:** 1 Nursing Department, Jaume I University, Avda Sos Baynat s/n, 12071 Castellón de la Plana, Spain; vchorda@uji.es (V.M.G.-C.); llagoste@uji.es (I.L.-R.); chillero@uji.es (M.J.V.-C.); cerveraa@uji.es (Á.C.-G.); 2 Department of Health in Castellón, Valencian Health System, Avda Benicassim, 128, 12004 Castellón de la Plana, Spain; claramonte_elo@gva.es

**Keywords:** virtual reality, older adults, active aging, cognitive impairment, accidental falls, acceptability

## Abstract

Background: Active aging is considered one of the most effective methods for a healthy aging process. There are numerous clinical practice guidelines that address this model and propose multiple strategies for its achievement through the improvement of motor and cognitive function. Virtual reality is emerging as a potential tool, with various modalities focused on promoting good health maintenance in older adults. The objectives of this review were to map the potential benefits of virtual reality for active aging and delve into adaptability and adherence in older individuals. Methods: A scoping review was conducted on studies published between 2013 and 2023 in English, Spanish, or Catalan, examining virtual reality interventions in older adults. The search was performed using the Medline, CINAHL, Scopus, and Web of Science databases. The methodological quality was assessed using CASPe and FLC 3.0 critical appraisal guidelines. The graphical data were reported narratively, grouping results based on the study characteristics and the impact of virtual reality. Results: The review process resulted in the inclusion of 22 articles out of the initial 459 following the application of the selection criteria. Most articles were randomized controlled trials (45.4%; *n* = 10), systematic reviews (40.9%; *n* = 9), observational studies (9%; *n* = 2), and pilot studies (4.5%; *n* = 1). The information was organized based on the virtual reality modality (immersive, non-immersive, and 360) and application area (motor, cognitive, and mental health). Conclusions: Virtual reality (both immersive and non-immersive) is a valuable tool for promoting physical exercise in older adults, helping to prevent recurrent accidental falls. It also yields positive results for cognitive stimulation in healthy older individuals, improving memory, depression, and mental health in those with cognitive impairment. Virtual reality is generally well-received by older adults, achieving high adherence rates.

## 1. Introduction

At present, one of the most relevant changes in developed countries is the progressive aging of the population and the increasing life expectancy [1]. In Spain, the average life expectancy is 83 years, a figure that has continuously increased since historical records began [2]. Furthermore, it is anticipated that by the year 2050, the Spanish population will be the world’s most aged, with individuals over 65 years old representing up to 30% of the total population [3].

This significant success has resulted from various implemented public policies favoring conditions that have led to an increase in the aging population [4]. Among them, a comprehensive care model based on health, safety, social participation, and continuous education for older adults throughout their life cycle, known as active aging [5], stands out. The World Health Organization [6] defines active aging as a “process of optimizing opportunities for health, participation, and security to enhance the quality of life as people age“.

Active aging proves to be a fundamental resource that assists individuals and communities in realizing their potential for quality of life and significance throughout their life cycle. It enables them to participate in society according to their needs, desires, and capabilities while providing support, protection, safety, and appropriate care when assistance is needed. The promotion of active aging requires fostering and balancing personal responsibility, intergenerational encounters and solidarity, and the creation of favorable environments that make healthy choices easy to make [7].

Among the means to achieve this paradigm of care for older individuals, the use of new technologies is increasingly prevalent. This demographic, becoming more familiar with digital tools, is integrating into an increasingly digitized society where new technologies can contribute to improving health by providing diverse alternatives aligned with their needs, interests, and conditions [8].

New technologies have become essential elements in the daily life of the older population, promoting their independence and contributing significantly to active aging. These technologies not only enable older individuals to navigate daily activities, communicate, and actively participate in society [9], but they can also offer specific benefits for improving their motor and cognitive functions. Interventions specifically designed for this demographic provide personalized experiences that stimulate cognitive function and may enhance motor skills, addressing key aspects of active aging [1,3,10]. Furthermore, the positive impact of new technologies on health is evident through specific applications on mobile devices, demonstrating effectiveness in improving cognitive and motor health in individuals aged 65 and older [8]. This technological integration not only empowers older adults in their daily lives but also enriches their physical and mental health, supporting active and healthy aging [10].

Currently, there are other technological models aimed at being tools for improving personal autonomy through cognitive and motor stimulation [3], combining with established conventional strategies for promoting healthy aging, such as physical exercise [9]. Among them are interactive experiences based on virtual reality with modalities that can be immersive, non-immersive, or highly immersive (also known as 360) simulation. Among the non-immersive experiences are interactive surfaces, which are technological interfaces that enable direct interaction between users and content through gestures, touch, and movements, projecting images onto the floor or wall [3]. Positive results have demonstrated that such experiences are beneficial for improving diverse aspects such as social isolation, pain, and psychomotor skills, among others [8].

Regarding the acceptance of the use of technological devices, older individuals tend to favorably view the utilization of those with which they are already familiar, despite any initial prejudice that may suggest otherwise. Given the digitization of society, older individuals are starting to perceive virtual reality as an increasingly common option. Moreover, these devices are becoming more prevalent in environments that provide care for older individuals, although literature focused on potential adverse effects resulting from their use is still scarce [9].

In light of the effects that new technologies have on this population, this scoping review has been conducted to systematically map the research carried out in this area and thus (1) explore potential benefits of interventions through virtual reality that enhance the active aging of older individuals, as well as (2) investigate the adaptability and adherence to these new technologies by this demographic. The present work is structured into several sections, including the introduction, where the topic and study objective are presented; the literature review, which addresses previous research and the theoretical framework; the methodology, which describes the research methods; the results, where the findings are presented and discussed; and the conclusions, which summarize the results and provide suggestions for future research.

## 2. Materials and Methods

### 2.1. Design

A scoping review was conducted following the Joanna Briggs Institute (JBI) methodology [11] for scoping reviews, which consists of four main components: (1) evidence generation in healthcare; (2) evidence synthesis; (3) evidence/knowledge transfer, and (4) research utilization. The review was also conducted according to the current version of PRISMA Extension for Scoping Reviews (PRISMA-ScR) [12] to locate, select, examine, and synthesize articles related to the effects of virtual reality on active aging in older individuals. As a preliminary step to conducting this review, a protocol was drafted and registered in November 2023 on the Open Science Framework, accessible through the link: http://dx.doi.org/10.17605/OSF.IO/YX73W (accessed on 14 December 2023).

Based on the intention to map the mentioned research field, two research questions were formulated using the PIO system. (1) Population (P): older individuals; Intervention (I): virtual reality; Outcomes (O): promotion of active aging through improvement in motor and cognitive function. The formulated research question was, therefore, How can virtual reality help improve motor and cognitive functions encompassed in active aging for older individuals? (2) Population (P): older individuals; Intervention (I): virtual reality; Outcomes (O): adaptability and adherence to virtual reality. What is the adaptability and adherence of older individuals to virtual reality interventions?

### 2.2. Eligibility Criteria

All studies had to be based on the use of virtual reality as a tool for improving cognitive and/or motor conditions in older individuals, whether institutionalized or not. Studies of all types of methodological design were included following the recommendations of Whittemore et al. [13], such as systematic reviews; randomized clinical trials; observational studies; and pilot studies exclusively focused on older individuals. Subsequently, compliance with the proposed inclusion/exclusion criteria was evaluated (Table 1).

The time frame of 10 years was chosen to encompass the existing literature since its implementation. Only articles with full-text access were included, as they allow for in-depth analysis, facilitating the achievement of the objectives outlined in this review. The language was restricted to English as the predominant scientific language, and Spanish and Catalan, the native languages of the authors of this review. Articles that did not fit within the conceptual framework of the study, i.e., those not directly related to the effects of virtual reality on active aging, were excluded.

### 2.3. Information Sources

To identify relevant studies related to the objectives of this scoping review, searches were conducted in December 2023 using the following databases: Medline, Scopus, Cumulative Index of Nursing and Allied Literature Complete (CINAHL), and Web of Science (WOS). These search tools were chosen for their international recognition and broad multidisciplinary coverage.

### 2.4. Search Strategy

To design a sensitive search in accordance with PRISMA-ScR parameters, the strategy focused on thematic terms that did not limit themselves to the specific controlled vocabulary of the databases. The identification of search terms was based on the clinical experience of two members of the working group (V.O.-M., E.C.-G.), who extracted keywords based on the formulated PIO clinical question.

These keywords were translated from natural language to controlled language terms in English using the Medical Subject Headings (MeSH) descriptor tool. The obtained terms were combined with the Boolean operators “AND” and “OR.” The search terms included aging (MeSH), healthy aging (MeSH), virtual reality exposure therapy (MeSH), exercise video game (MeSH), and virtual reality (MeSH). Additionally, to broaden the search based on the review objectives, natural language terms such as interactive floor game projection, interactive wall game projection, and interactive surfaces were used.

A general search strategy (Table 2) was designed by two team members (V.O.-M., E.C.-G.), later reviewed and agreed upon by all the researchers (V.M.G.-C., A.C.-G., M.J.V.-C, I.L.-R.). It was used for all consulted databases, with necessary adaptations based on each database’s characteristics, aiming to maintain consistency. Additionally, other complementary, more specific search strategies were designed to explore potential uses of virtual reality in active aging through exercise video games or interactive surfaces.

The search strategy underwent a review process conducted by two reviewers (V.O.-M., E.C.-G.). The Peer Review of Electronic Search Strategies (PRESS) [14] checklist was used to ensure the quality and validity of the search strategy, as it enhances the reliability and reproducibility of the search methodology. It was confirmed that both reviewers obtained the same results after conducting searches in each database, bolstering the confidence in the integrity of the search process.

### 2.5. Selection of Sources of Evidence

Two researchers (V.O.-M., E.C.-G.) independently assessed the titles and abstracts of the retrieved articles in the initial selection to verify their relevance to the review topic. Subsequently, each reviewer independently conducted a full reading of the selected documents to confirm whether they met the selection criteria and to perform a critical evaluation of the methodological quality of the studies. Articles were categorized based on their methodological quality, with both reviewers considering the articles to be of high quality to be selected. The results of both reviewers were compared (V.O.-M., E.C.-G.), and the discrepancies were discussed with the rest of the researchers (V.M.G.-C., A.C.-G., M.J.V.-C, I.L.-R.) until a consensus was reached. This procedure was established as the tools used to assess methodological quality did not provide cutoff points to determine the level of methodological quality.

### 2.6. Data Extraction Process

The data were extracted using a Microsoft Excel (version 16.80) spreadsheet. Two reviewers (V.O.-M., E.C.-G.) separately performed the extraction, and later compared the results and discussed them to ensure data uniformity and consistency. The extracted data from the articles included general study information (e.g., authors, year), sample description (e.g., size, age), intervention description (e.g., intervention content, duration), control description, measurement instruments, and primary outcomes.

### 2.7. Methodological Quality Assessment

For the methodological quality assessment, the Critical Appraisal Skills Programme in Spanish (CASPe) [15] and the Web 3.0 Platform for Critical Reading Sheets (FLC 3.0) [16] were used. Each study was independently rated by the authors as high or low quality, with those classified as having low methodological quality being excluded. To address the concerns raised among the reviewers involved in this process (V.O.-M., E.C.-G.), consensus was sought through a joint meeting involving all the researchers. It was decided to numerically set the cutoff point between articles of low or high quality at 7 on a scale from 0 to 8. As there is no consensus on this aspect, the authors chose to assign a high value to the cutoff point, aiming to ensure the highest possible methodological quality.

Randomized controlled trials and systematic reviews were evaluated using the sheets provided by the Critical Appraisal Skills Programme (CASPe) [15]. The scoring range for both types of studies is from 0 to 8, as the last two additional questions relate to the overall study outcome and invite the reviewer to express their personal opinion. In this review, the methodological quality of articles was assessed using this tool, and only articles that scored 7 or higher were included.

To assess observational studies and pilot studies, templates from the Web 3.0 Platform for Critical Reading Sheets (FLC 3.0) [16] were used. In order to standardize the evaluation process, a similar range to the CASPe tool was offered, also setting the cutoff at 7 points.

### 2.8. Synthesis of Results

Given the heterogeneity of the included studies in terms of intervention characteristics, study duration, and outcome measures, the data were summarized narratively, grouping results according to study characteristics and the impact of virtual reality on active aging. Subcategories of primary and secondary outcomes of interest were identified. Additionally, differences and similarities between significant and non-significant findings were analyzed in the context of the intervention, population characteristics, and other study features.

## 3. Results

### 3.1. Study Selection

**Figure 1:**
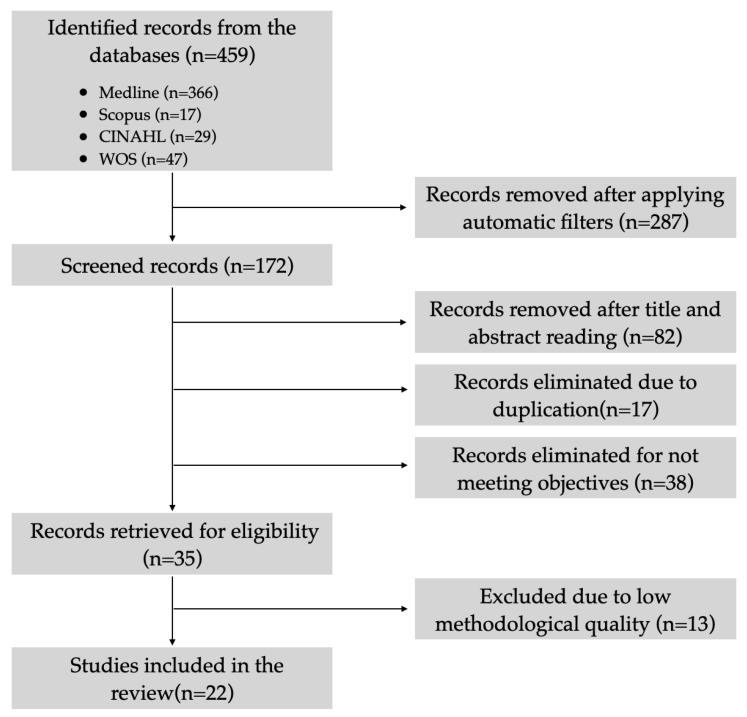
PRISMA diagram of the definitive article selection process.

A total of 459 articles were initially retrieved without applying the inclusion and exclusion criteria, primarily sourced from the Medline database (79.7%; *n* = 366), followed by Web of Science (10.2%; *n* = 47), CINAHL (6.3%; *n* = 29), and Scopus (3.7%; *n* = 17). After applying filters, this number was reduced by 37.4% (*n* = 287), resulting in 172 articles. Subsequently, duplicate articles were removed (3.7%; *n* = 17), and titles and abstracts were examined, leading to the elimination of 82 articles (17.8%) that were not closely related to the topic under consideration. Following this, the remaining articles underwent a full-text review, and 38 articles (8.3%) were excluded for not aligning with the review objectives. For the final 35 articles (7.6%), a critical reading was conducted, resulting in the exclusion of 13 articles (2.8%) due to low methodological quality. Finally, a total of 22 articles (4.8%) were included in this scoping review [17,18,19,20,21,22,23,24,25,26,27,28,29,30,31,32,33,34,35,36,37,38]. The PRISMA flow diagram illustrating the selection process is presented in Figure 1.

### 3.2. Characteristics of the Studies

Among the included studies, ten (45.4%) were conducted in Asia (China, Taiwan, Iran, and South Korea), seven (31.8%) in Europe (Spain and the Netherlands), and five (22.7%) in the Americas (Colombia, the USA, Canada, and Brazil). All studies were published between 2013 and 2023, with the majority being recent publications (between 2020 and 2023). In terms of study types, ten (45.4%) were randomized controlled trials, nine (40.9%) were systematic review studies, two (9%) were observational studies, and only one (4.5%) article was a pilot study.

### 3.3. Critical Assessment of Methodological Quality

In the final stage of the selection process, a critical assessment of methodological quality was conducted on the 35 (7.6%) selected articles following the selection criteria. Using the Critical Appraisal Skills Programme in Spanish (CASPe) [15] and the Web 3.0 Platform for Critical Reading Sheets (FLC 3.0) [16] evaluation tools, a total of 22 (4.8%) articles scored seven points or higher, while thirteen articles with lower scores were excluded due to a low methodological quality (ten received a score of five points, while two received six points, and one received three points).

### 3.4. Results of the Synthesis

Table 3 presents the results of the selected articles (country of origin and study design), along with a description of the objective, participants, type of virtual reality used, worked dimension, adaptability to the intervention, and main conclusions.

The information about the participants included whether they were older adults with or without cognitive impairment or if they had a history of cerebrovascular issues, as well as whether they were institutionalized. Of the studies, 40.9% (*n* = 9) were studies based on interventions with older adults residing in the community without cognitive impairment, while 27.2% (*n* = 6) were studies with institutionalized participants; 18.1% (*n* = 4) of the studies focused on older adults with cognitive impairments, whether living in the community or in residential centers. The remaining 13.6% (*n* = 3) were studies with participants who were older adults with diverse backgrounds, such as cerebrovascular or psychiatric issues, or problems with balance.

The type of virtual reality on which the study was based was also recorded, distinguishing between immersive, non-immersive, or 360-degree virtual reality. The majority of interventions (54.5%; *n* = 12) used immersive virtual reality through virtual reality goggles. Among the 31.8% (*n* = 7) of studies using non-immersive interventions, some were based on exergames or interactive surfaces. Lastly, 9.1% (*n* = 2) of the studies used both immersive and non-immersive reality, and only 4.5% (*n* = 1) were based on 360-degree experiences.

The interventions aimed to provide benefits in various areas of active aging, and we differentiated between those addressing cognitive, motor, and mental health aspects. Most interventions focused exclusively on motor aspects (49.1%; *n* = 9) or combined motor and cognitive aspects (31.8%; *n* = 7). To a lesser extent, there were experiences focused exclusively on cognition (9.1%; *n* = 2), or cognition combined with psychiatric issues (13.6%; *n* = 3) or with motor and psychiatric issues (4.5%; *n* = 1).

Similarly, given the user profile, information was extracted about the adaptability of these subjects to virtual reality interventions, as well as the degree of adherence. While the vast majority of studies selected for the review (77.2%; *n* = 17) did not report on this, the 22.7% (*n* = 5) that did showed very high adherence, with no significant adverse effects reported that hindered the adaptability of most participant subjects. In the reported cases, the majority of adverse side effects were mild dizziness and fatigue.

## 4. Discussion

The present scoping review was undertaken with the primary objective of investigating the possibilities that virtual reality can offer for promoting active aging in older adults, focusing on two areas of this concept that foster it the most: cognitive function and motor function [1,3,9]. Additionally, it aimed to explore the adaptability and adherence of older individuals to this type of new technology.

This work is grounded in its distinctive approach, characterized by the inclusion of recent studies, a critical analysis revealing emerging patterns and gaps in the literature, and an updated synthesis. In contrast to other consulted reviews [19,20,21,22,23,24,25], our review adopts a comprehensive perspective by addressing all modalities of virtual reality. This broad approach aims to obtain a holistic view of the use of virtual reality in active aging, aligning with the considerations of other authors [26,27], who highlight the existence of a wide variety of works focused on different virtual reality environments. By including all modalities, from immersive to non-immersive, we seek to provide a more comprehensive and updated understanding of the applications and potential benefits that virtual reality can bring to the promotion of active aging.

Furthermore, it is important to note that this review has been conducted exclusively considering the most recent evidence in this field, in contrast to other consulted reviews that adopted a broader time frame [21,22,23,24,27]. This decision aims to provide an up-to-date and accurate view of the most recent trends and advances at the intersection of virtual reality and active aging. This work goes beyond mere description of existing literature and offers practical recommendations for the effective implementation of virtual reality interventions in active aging programs. These recommendations seek to provide useful guidance for professionals, researchers, and planners looking to integrate virtual reality effectively into active aging strategies.

### 4.1. Impact of Virtual Reality on Motor Function in Older People

Aging and its associated diseases can lead to negative changes in the motor skills necessary for mobility, resulting in decreased participation in the community, increased need for assistance, and decreased quality of life. Furthermore, they often lead to an increased risk of falls, related to a loss of balance and decreased muscle strength and motor responses. In contrast, physical exercise has been recognized as an important strategy in the promotion of healthy aging [36], and an effective method in the prevention of falls.

The results found in this review show that there is a majority consensus that physical exercise programs based on virtual reality are capable of improving essential aspects for the prevention of falls in older adults, such as balance and muscle strength [17,20,21,26,27,28,29,36]. Despite this, other authors [19] highlight the still scarce and heterogeneous evidence available in this regard, which makes it necessary to be cautious with the statements obtained in the studies carried out to date. In general, all the results evaluated are based on randomized clinical trials in which an experimental group on a physical exercise program using virtual reality was compared with a control group in which a traditional program was used (and sometimes these have been compared with a third non-intervention group).

These programs are not only beneficial in these aspects but can also help the elderly to lose their fear of recurrent falls, as mentioned by Zahedian-Nasab et al. [28] in their randomized clinical trial, where they observed how balance exercises worked through virtual reality were able to reduce the fear of falling again in elderly people who had recently experienced a fall. This aspect coincides with Martínez Montilla et al. [17] and is directly linked to an increase in independence in daily living activities.

Physical fitness is essential for the prevention of falls in older adults, which is an aspect that virtual reality can improve through an increase in muscular balance [21,26,29]. In this sense, an adequate muscular grip in the hands is essential for the proper handling of the technical aids used by the elderly for fall prevention (canes, crutches, walkers, etc.). Physical exercise programs based on virtual reality may be able to increase the grip strength in the hands, according to the results obtained [27].

For the maintenance of balance when standing and during gait, knee locking in extension is necessary for quadricep muscle contraction. Ren et al. [20] concluded in a systematic review that virtual reality interventions could improve knee extension muscle strength, improving physical function and consequently minimizing falls, with greater strength than seen with traditional exercise interventions. Similarly, another trial [29] focused on the muscle function of the quadriceps and hamstring muscles, and showed positive values in a combined intervention strategy versus traditional exercises, exclusive virtual reality training, and the control group. These authors consider the combination of virtual reality and traditional exercises as essential to obtain positive results in their trial.

Therefore, there is some disparity in the approach to interventions, since positive results have been found in the gain of muscle strength and balance both in programs exclusive to virtual reality [20,21,26,28] and in programs that combine traditional exercises with virtual reality [25,29]. In relation to this aspect, a recent systematic review [25] set out to investigate which type of intervention would be more effective in this population: virtual reality specifically designed to improve balance, non-immersive virtual reality based on exergames, or conventional treatment. It was concluded that exergames solved the problems posed with greater affinity and could therefore better prevent falls in older people.

Another non-immersive virtual reality technology used in this field is interactive surfaces. Whether the activity is on the floor or on the wall, it is emphasized that the projection helps users to be more physically active. The studies by Braun et al. [34] and Luyten et al. [35] aimed to encourage physical activity in the elderly through interactive surfaces that generated this interaction without the intervention of a therapist, obtaining good results since they were attractive to participants. The elderly spontaneously engaged physically in the interactive surface without explicit (verbal) instructions or prompting. In addition, the results of a Spanish study show that this technology is capable of producing sufficient physical, cognitive, and socioemotional stimulation to be used as a stimulation tool for the elderly [15].

Nevertheless, the choice of the type of content to be projected and the role of the activity provider are fundamental for the intended experience to be comfortable and effective for the elderly [35]. The participation, therefore, of a therapist in the development of these activities is essential, not only for the control and management of possible adverse effects, but also because if they motivate and explain the experience to the participants, the results will be more satisfactory [15]. With all this considered, we could help to mitigate the barriers described to the use of new technologies for the promotion of active aging [15].

Regarding the minimum duration of interventions focused on balance and muscle strength (with an impact on the prevention of falls in older adults), there is a disparity of opinion, since some articles [17,20,28] state that the benefits begin to be apparent around the fifth week, while studies such as that of Campo-Prieto et al. [27] and others [29,36] found that the gain in balance and strength was obtained from the tenth week onward. Only Liao et al. [32] found benefits from week 12, probably because the entire population of their study consisted of elderly people with cognitive impairment, unlike the rest of the studies in which this fact was not proven. Finally, the mean duration of the virtual reality training sessions was about 40 minutes [20,28,29], three times per week [20,29].

### 4.2. Impact of Virtual Reality on Cognitive Function in Older People

Like balance and motor functions, cognition is another of the abilities generally affected—to a greater or lesser extent—in older people. The results show how specific virtual reality interventions in this field generally focus on memory and executive functions, obtaining promising results both in a healthy or non-typical elderly population [21,30], and in those elderly with a diagnosis of mild cognitive impairment [22,32]. On the other hand, studies such as that of Park et al. [31] found no improvement in cognitive function after three months of training in elderly people with mild cognitive impairment. Lin et al. [18] and Yen et al. [23] showed a moderate impact on cognition, although they did obtain significant results on depression, concluding that “virtual reality games can be an interesting strategy for active aging and good maintenance of mental state” [23].

It seems that interventions through virtual reality are able to improve neuropsychiatric symptoms present in many older people [24] due to the natural aging process being accompanied by cognitive impairment; this is also true in those with severe and advanced stage dementia, as concluded by Sanchez et al. [16] in their trial. In this trial, an intervention group using virtual reality in a multisensory stimulation environment and a control group with a traditional individualized intervention were compared for 16 weeks. After eight weeks, positive results were already found in the mental state of the participants in the experimental group. However, the importance of maintaining the stimulation with virtual reality over time to ensure the maintenance of the benefits achieved is emphasized; this criterion was also recommended by other authors [23,25].

Therefore, virtual reality can be an ally for cognitive stimulation, but it also seems to be a tool for detecting cognitive impairment in older adults. In fact, the review by Skurla et al. [24] found that tests and scales based on virtual reality demonstrate a validity comparable to some cognition assessments traditionally performed on paper, although more work is needed to refine the diagnostic specificity compared to that of the usual methods.

Another aspect to highlight is the probable relationship between combined virtual reality interventions (physical exercise and cognitive stimulation) and the achievement of positive results for cognitive function. This hypothesis is raised in articles outside this review [37,38], where it was concluded that physical exercise plays a key role in the prevention of dementia and may have positive effects on cognitive function in older people.

Although some virtual reality games are not designed to improve cognitive function initially, this effect may be due to the unique neurobiological contributions of each form of intervention (both physical and cognitive). According to Liao et al. [32], combined interventions may maximize these underlying neurobiological mechanisms and processes, resulting in a synergistic or additive effect that promotes neurogenesis and cell survival, such that the combination of physical exercise and cognitive work with virtual reality is able to elicit prefrontal cortex activation in older people with mild cognitive impairment. In this way, global cognitive abilities [15,18,21,22], memory [22,23], and depression [18,23] can be improved.

### 4.3. Acceptance and Adherence of Older People Using Virtual Reality Activities

A common and apparently erroneous belief about the use of virtual reality is the lack of acceptance or the frequent occurrence of discomfort in the elderly. According to the results obtained, virtual reality is considered an enjoyable activity, regardless of whether the elderly person is healthy or cognitively impaired [28]. According to Campo-Prieto et al. [36], who conducted a trial with immersive virtual reality for 10 weeks on a nonagenarian population, no adverse effects were found, and it was considered an effective and feasible technology.

In general, virtual reality is considered an attractive experience, as it does not often cause discomfort or adverse effects in the elderly [27,36], although dizziness or fatigue may occasionally occur [31]. These symptoms, known as “cybersickness,” are attributed to the discrepancy between the visual signals of virtual reality and internal sensory signals. Factors such as the quality of the experience and individual sensitivity can influence the onset of motion sickness. Although it is an occasional effect, recent research aims to understand and mitigate these side effects [31].

However, some skill is required for the use of virtual reality, so as to avoid injury: a certain level of cognition and physical fitness is needed when playing exergames [21]. In this sense, the role of the therapist or caregiver during the virtual reality activity is essential to avoid the possible occurrence of adverse effects, as well as to promote the participation of the elderly [35], among other reasons, because the majority of the included studies did not consider the digital competence level of the participating subjects, and this aspect can be crucial for the user to adapt well to its use [19].

Another generally incorrect aspect is the lack of acceptance of virtual reality activities in the elderly, since according to the findings of this review, adherence rates are particularly high [19,27], sometimes exceeding rates of 90% of cases [31]. Therefore, it can be affirmed that the use of virtual reality in the elderly is feasible, safe, and tolerable [15,19,21,27,31,36].

### 4.4. Immersive Virtual Reality Versus Non-Immersive Virtual Reality

Although non-immersive virtual reality has been implemented in the field of health for several years, there are recent trials and systematic reviews addressing its use in the context of aging. As reported by Skurla et al. [26], their review found a wide variety of virtual reality environments; nearly all the studies they analyzed used a computer screen and a mouse to interact with virtual scenes (non-immersive modality), although immersive virtual reality headsets were more common among articles published more recently (from 2020 onward). Similarly, other works published in the last three years [20,22,23,25] focused solely on non-immersive virtual reality, as this modality remains the most commercially accessible today. There are also studies suggesting that these types of non-immersive exercise games have the advantages of exciting game content and instant feedback, providing real exercise experiences and effectively stimulating sensory, cognitive, psychological, and motor functions in older individuals [20,23].

These reviews, obtained following a similar criterion to the present review, either did not set a time limit in the database searches or set it within the last 10 years. This may be attributed to the continued novelty of using virtual reality in the field of active aging, regardless of the modality employed, although with a clear trend in recent years toward immersive technologies, increasingly advanced and with greater capabilities. However, as noted by other authors [26], Doré et al. agree that despite this trend, more studies are still needed to draw stronger conclusions about the effects of this modality on older individuals [21].

They also emphasize that the upgrading of immersive devices compared to more primitive and lower-quality ones has consistently reduced the occurrence of side effects in older individuals, especially those derived from poor oculocervical adaptation that can consequently induce dizziness or cybersickness [21]. However, non-immersive virtual reality may be better accepted by older individuals due to its lower sensory intensity, lower susceptibility to motion sickness, greater ease of adaptation, less demanding technical requirements, focus on calmer content, and lower likelihood of social disconnection [39,40].

#### 4.4.1. Limitations

The main limitation of this review lies in the heterogeneity found among the included studies. The limited availability of detailed data, such as differences in study designs, sample populations, evaluated interventions, and outcome measurements in some studies, hindered the interpretation of the results. As indicated by some authors [41], this constraint can occasionally occur in scope reviews, making the execution of a heterogeneity analysis challenging. This limitation may have introduced some bias that distorts the results; hence, we encourage the recommendation for future reviews or meta-analyses to conduct more specific studies, allowing for a more precise assessment of variability among the studies. This will assist in confirming the existence of biases that may influence the overall conclusions.

Likewise, the search did not distinguish between healthy or non-typified older adults and older people with dementia, which is an aspect that may also have biased the interpretation of the results. Finally, it has been difficult to find studies dealing with interactive surfaces, which makes it necessary to be cautious with the extrapolation of these findings.

#### 4.4.2. Utility and Future Research

The importance of this review lies in raising awareness of the usefulness of virtual reality in physical and cognitive exercise training for the elderly in order to promote healthy aging. In light of the results, it can be seen that few current research studies have delved into this fact, which should serve as a stimulus to continue working on this area to encourage new studies that delve deeper into the possible positive effects of this new technology.

Similarly, the scientific community is encouraged to increase research in this area, delving into the underlying mechanisms of the observed effects and further exploring the practical applications of virtual reality in active aging. It is suggested that future studies focus on more specific intervention protocols, considering the diversity of the older population and adapting virtual reality technologies to individual needs. Likewise, conducting a meta-analysis would add value by providing a quantitative and objective synthesis of available evidence, enhancing the understanding of the studied phenomena and offering a more robust foundation for decision making.

This review aims not only to highlight the proven benefits of virtual reality in active aging but also to stimulate ongoing research and promote wider adoption of these technologies in health and social well-being settings.

## 5. Conclusions

The results reveal that interventions designed to improve balance and muscle strength in older individuals through the use of virtual reality are effective, generating an additional positive effect in preventing accidental falls in this population. According to the literature, these benefits are achieved through virtual reality exercise programs consisting of sessions lasting approximately 40 minutes for three days a week, although there is no consensus on the total duration of these programs required to achieve improvements, all of which are typically longer than five weeks on average.

Similarly, interventions using virtual reality to stimulate higher cognitive functions, such as memory, depression, and mental health, show positive effects in older individuals with mild cognitive impairment. In those without identified cognitive impairment, these interventions succeed in improving brain function, especially when physical exercise is combined with cognitive stimulation.

Regarding the acceptance of these interventions by older individuals, the results indicate that virtual reality, regardless of the modality used, is generally well-received and usually does not produce significant adverse effects that hinder its use, achieving very high adherence.

In light of the results obtained in this scoping review, it is concluded that virtual reality can achieve positive outcomes in areas related to aging. It positions itself as a valuable tool for healthcare professionals in promoting active aging in older individuals who are living in the community or institutionalized. It is suggested that future research focus on optimizing the duration and frequency of interventions, as well as exploring new areas of application for virtual reality in the context of aging.

## Figures and Tables

**Table 1:** Article selection criteria.

Inclusion Criteria	Exclusion Criteria
Publications from the last 10 years (2013–2023) Articles with full-text access Articles written in English, Spanish, or Catalan Studies on the applicability of virtual reality in the elderly population Studies linking virtual reality with active aging Articles meeting high methodological quality criteria	Duplicate entries Studies not available in full text Studies unrelated to the topic Publications with low methodological quality

**Table 2:** General search strategy.

((“virtual reality”(MeSH Terms) OR (“virtual”(All Fields) AND “reality”(All Fields)) OR “virtual reality”(All Fields) OR “virtual reality”(MeSH Terms)) AND (“healthy aging”(MeSH Terms) OR (“healthy”(All Fields) AND “aging”(All Fields)) OR “healthy aging”(All Fields) OR “healthy aging”(MeSH Terms))) AND ((y_10(Filter)) AND (ffrft(Filter)) AND (catalan(Filter) OR english(Filter) OR spanish(Filter)))

**Table 3:** Additional information on the reviewed articles.

Reference, Country, Design	Objective	Subjects, Type of Virtual Reality	Worked Dimension(s)	Good Acceptability?	Conclusions
Gómez, F.E. et al., 2019 [17] Spain Observational study	Development and validation of an exercise integrating automatic recognition of the elderly person’s position	Elderly adults Non-immersive virtual reality (interactive surface)	Cognition Motor skills	Yes	The approach to the exercise, both in terms of the theme and the mode of interaction, may be well received by the elderly and thus contribute to health promotion. It can serve as the foundation for the development of new exercises implementing various interactive floor modalities and training different technical characteristics
Sánchez, A. et al., 2016 [18] Spain Randomized clinical trial	Compare the effect of a group multisensory stimulation environment with traditional individual sessions	Elderly individuals with cognitive impairment 32 subjects 360 experience	Cognition Mental health	No report	The intervention group patients demonstrated a significant improvement in the Neuropsychiatric Inventory and scores on the Bedford Alzheimer’s Nursing Severity Scale compared to the activity group. All participants showed improvement during the intervention in the aggressive behavior factor of the Cohen-Mansfield Agitation Inventory and the total score, with no significant differences between the groups. Multisensory stimulation may have better effects on neuropsychiatric symptoms and dementia severity compared to individual activity sessions
Martínez-Montilla, L.A. et al., 2023 [19] Colombia Systematic review	Determine the effectiveness of virtual reality in balance training	Older adults	Motor skills	No report	The benefits presented by the studies were related not only to balance but also to the fear of falling, reaction time, gait, physical fitness, independence in activities of daily living, muscular strength, and even quality of life
Lin, C. et al., 2023 [20] China Systematic review	Assessing the effectiveness of virtual reality games on cognition, mobility, and emotion in elderly patients	Older adults with a history of cerebrovascular events 1331 subjects Non-immersive virtual reality	Cognition Motor skills Mental health	No report	Training in sports games, especially with virtual reality setups, had a positive impact on improving cognitive performance, mobility, and emotional state in stroke patients compared to a control group. Although the improvement in cognitive capacity was relatively modest, the effect on enhancing physical activity and reducing depression was evident
Doré, B. et al., 2023 [21] Canada Systematic review	Synthesizing the literature on the acceptability, feasibility, and effectiveness of virtual technology for promoting physical exercise	Older adults 1853 subjects (54 studies) Immersive virtual reality	Motor skills	Yes	Virtual reality seems to be well accepted by older individuals, and its use with this population is feasible. However, more studies are needed to conclusively determine its effectiveness in promoting exercise among older people
Ren, Y. et al., 2023 [22] China Systematic review	Exploring the effects of virtual reality on physical function, balance, and falls	Elderly individuals with impaired balance 2404 subjects (23 studies) Non-immersive virtual reality	Motor skills	No report	Virtual reality interventions can assist older individuals with balance impairment in overcoming traditional sports obstacles and improving physical function, balance, and minimizing falls. A virtual reality intervention based on balance training is more effective in balance recovery and fall prevention compared to the gaming program
Chen, P.J. et al., [23] 2023 Taiwan Systematic review	Analyze the effectiveness of physical training using virtual reality	Institutionalized older adults 482 subjects (12 studies) Non-immersive virtual reality	Cognition Motor Skills	Yes	Exercise games can enhance the balance capacity of older adults residing in senior care centers
Yan, M. et al., 2022 [24] China Systematic review	Evaluate the effects of cognitive and physical interventions based on virtual reality on cognitive function	Elderly individuals with cognitive impairment 188 subjects (7 studies) Immersive virtual reality	Cognition Motor skills	No report	This meta-analysis examined the potential rehabilitative effects of combined cognitive and physical virtual reality interventions for older adults with mild cognitive impairment
Yen, H.Y. et al., 2021 [25] Taiwan Systematic review	Explore the effectiveness of virtual reality exercises in enhancing cognition in older adults	Older adults 18 studies Non-immersive virtual reality	Cognition Mental health	No report	Virtual reality exercises may offer potential positive influences on cognition, memory, and depression in older adult populations. Virtual reality games could be an interesting strategy for active aging and maintaining good mental health
Skurla, M.D. et al., 2022 [26] USA Systematic review	Determine the levels of evidence for the use of virtual reality in clinical settings	Older adults with psychiatric history 2524 subjects (55 articles) Immersive and non-immersive virtual reality	Cognition Mental health	No report	The existing evidence provides support for virtual reality as a tool for the detection and treatment of cognitive impairment in older adults. Virtual reality demonstrates a validity comparable to some paper-based assessments for detecting cognitive issues
Corregidor-Sánchez, A.I. et al., 2021 [27] Spain Systematic review	Compare the effectiveness of rehabilitation programs using three different types of virtual reality-based interventions to enhance mobility in older adults	Older adults 568 subjects (18 studies) Immersive and non-immersive virtual reality	Cognition Motor skills	No report	Virtual reality is an effective intervention for improving functional mobility in older individuals compared to conventional therapy. Non-specific virtual reality has proven to be more effective than specific virtual reality
Kim, S.H. et al., 2022 [28] South Korea Randomized clinical trial	Examine the effect on balance of a virtual reality intervention	Institutionalized older adults 34 subjects Non-immersive virtual reality	Motor Skills	No report	Virtual reality and motor imagery are effective training methods to prevent physical weakness in institutionalized older adults
Campo-Prieto, P. et al., 2022 [29] Spain Randomized clinical trial	Explore the feasibility and effects of a virtual reality program on the physical functions of institutionalized older adults	Institutionalized older adults 24 subjects Immersive virtual reality	Motor skills	Yes 100% adherence	The findings indicate that virtual reality interventions are a feasible method for implementing a personalized exercise program and an effective way to enhance physical function in the target population. Improvements were observed in balance, gait, and grip strength
Zahedian-Nasab, N. et al., 2021 [30] Iran Randomized clinical trial	Investigate the impact of Xbox Kinect-based virtual reality exercises on balance and fall prevention	Institutionalized older adults 60 subjects Non-immersive virtual reality	Motor skills	No reports	Six weeks of virtual reality balance exercises could enhance balance and fall prevention among older individuals living in nursing homes
Sadeghi, H. et al., 2021 [31] Iran Randomized clinical trial	Compare the effectiveness of balance training among conventional treatment, virtual reality, and combined exercise	Older adults 64 subjects Immersive virtual reality	Motor skills	No reports	All treatment modalities improved leg strength, balance, and functional mobility compared to the control group. Combined exercise resulted in significantly greater improvements than the other intervention groups and was more effective in preventing falls
Wais, P.E. et al., 2021 [32] USA Randomized clinical trial	To observe whether a virtual reality spatial orientation intervention can enhance long-term memory capacity	Older adults 48 subjects Immersive virtual reality	Cognition	No reports	The results demonstrated a post-treatment gain in long-term memory capacity for the virtual reality intervention with Labyrinth, in comparison to the control group (placebo)
Park, J.H. et al., 2020 [33] South Korea Randomized clinical trial	Check if a culturally based virtual reality program is feasible and well-tolerated in older adults	Older adults with cognitive impairment 22 subjects Immersive virtual reality	Cognition	Yes 91.5% adherence	While the 12-week culturally based virtual reality training program did not improve cognitive function, the findings revealed that the culturally based virtual reality training program was feasible and well tolerated by participants with cognitive impairment
Liao, Y.Y. et al., 2020 [34] Taiwan Randomized clinical trial	Investigate the effects after 12 weeks of physical and cognitive training with virtual reality	Older adults with cognitive impairment 34 subjects Immersive virtual reality	Cognition Motor skills	No reports	Virtual reality-based physical and cognitive training improves cognitive function, instrumental activities of daily living, and neuronal efficiency in older adults with mild cognitive impairment
Bezerra, I.M.P. et al., 2018 [35] Brazil Randomized clinical trial	Evaluate if a task practiced in virtual reality can promote better performance than its real-world equivalent	Older adult 65 subjects Immersive virtual reality	Motor skills	No reports	The virtual environment provides an improvement in performance with a short-term motor learning protocol on a time-matched task in older adults
Braun, S.M., 2015 [36] The Netherlands Observational study	Determining how older adults in residential settings respond to the use of interactive floor surfaces	Institutionalized older adults 58 subjects Non-immersive virtual reality (interactive floor surface)	Cognition Motor skills	No reports	Interactive floor surface technology can be a promising tool for nursing home residents to stimulate physical activity
Luyten, T. et al., 2018 [37] The Netherlands Pilot study	Determining how older adults in residential settings respond to an interactive art installation	Institutionalized older adults with cognitive impairment 10 subjects Non-immersive virtual reality (interactive floor surface)	Cognition Motor skills	No reports	“VENSTER” evokes responses in residents of nursing homes with dementia, illustrating the potential of interactive artworks in the nursing home environment
Campo-Prieto, P. et al., 2022 [38] Spain Randomized clinical trial	Explore the usability and effects on balance of a virtual reality exercise program	Older adults 12 subjects Immersive virtual reality	Motor skills	Yes 100% adherence	Virtual reality is a feasible and effective method to improve balance and reduce the risk of falls in elderly individuals of advanced age

## Data Availability

Not applicable.
